# Supplementary material for: Probabilistic models of individual and collective animal behavior
Source: PLoS One. 2018 Mar 7;13(3):e0193049. doi: 10.1371/journal.pone.0193049 (PMC5841767; doi:10.1371/journal.pone.0193049)
Supplement: S1 Code — (ZIP) [file pone.0193049.s001.zip › S1_code/readme.rtf]

This folder contains the implementation of the inference method in matlab, as described in the paper. We provide two basic applications of the method on toy models: one in the model of ant motion, the other in the bacterial chemotaxis, and one application to the fish motion. The code in the first two cases is able to do two things: 1. Generate stochastic trajectories using the Gillespie method and the underlying stochastic model.2. Perform inference of the transition rates.The inference uses a conjugate gradient method for the minimisation of the log-likelihood. We use the library minFunc 2012, capable of a large-scale optimization. All examples contain a “masterfile” that runs all components of the program, including initialization of parameters, stochastic simulation, inference and visualization tools.SINGLE ANT:Inference uses the tiling functions and is based on computing two sets of summary statistics:	- number of transitions per each bin of the space	- total time spent in each binWe simulate many trajectories of medium length rather than one long trajectory to reduce the memory load of the computation. The results do not depend on this choice.MULTIPLE ANTS:Inference uses the tiling functions and is not based on summary statistics. Instead, the full trajectories are used for the inference of the transition rates. We use the fact that the transition rates are identical for all ants, therefore we use the trajectories of all ants and their response to other individuals to obtain more information about the behavior.BACTERIAL CHEMOTAXIS:The simulated trajectories of the tumble-and-run motion are annotated using two models:	- microscopic model with three deterministic behavioural states: tumble left/right, run,	- macroscopic model with one deterministic state (run) and one stochastic state (tumble)The second case is implemented assuming that the transitions between tumble left and tumble right are known. However, our numerical experiments suggest a negligible dependence on these parameters.FISH MOTION:Download the fish data from the following link: https://github.com/harpazone/Zebrafish-swimming-dataThe code uses four data files on the motion of two fish:2fish(22FD)coor.mat2fish(22FD)Wall.mat2fish(23FD)coor.mat2fish(23FD)Wall.matRunning the main file (inference_main.m) performs segmentation of the fish trajectories to three states: passive, active left, and active right. The model studies dependence of the transition rates on three kinematic variables: velocity (V), wall distance (W), and mutual distance/alignment of the two fish (D). The code performs inference of the fish motion based on seven models of different complexity, based on which kinematic variables it includes: VWD, VW, VD, WD, V, W, D. It returns the figures of inferred transition rates as well as the log-likelihood of each model.
